# Coronary Arteriovenous Fistula Secondary to Percutaneous Coronary Intervention of Chronic Total Occlusion 

**DOI:** 10.1155/2013/706820

**Published:** 2013-06-20

**Authors:** Seshasayee Narasimhan

**Affiliations:** ^1^Manning Rural Referral Hospital, Cardiovascular Division, Department of Medicine, Hunter New England Health Services, Taree, NSW 2430, Australia; ^2^Conjoint Senior Lecturer, University of New England, School of Rural Medicine, Armidale, NSW 2351, Australia; ^3^Mazankowski Alberta Heart Institute, University of Alberta, Edmonton, AB, Canada

## Abstract

This is a case report of a 61-year-old female presenting with ongoing chest pain in the setting of an NSTEMI with lateral ST-T changes. On attempting to open the left circumflex (LCX), it resulted in a proximal LCX dissection. The patient remained stable with no further chest pain. She was treated with IV Eptifibatide for 48 hours and restudied in 72 hours. Repeat coronary angiography showed a marginally improved proximal dissection plane with a coronary AV fistula. She was managed conservatively and discharged with a non-invasive assessment in 8 weeks. The patient had a negative stress echocardiogram and was managed with maximal medical therapy.

## 1. Introduction

Coronary AV fistulae are uncommon and bypass the myocardial capillary network and connect a coronary artery to another vessel or cardiac chamber. The primary etiology of coronary AV fistula is congenital and 0.25% is iatrogenic [1, 2]. Iatrogenic coronary AV fistulas are seen after acute myocardial infarction, aortic valve replacement, coronary angioplasty, coronary artery bypass graft surgery (CABG), endomyocardial biopsies, and thoracic trauma [3–11]. Given that iatrogenic coronary AV fistula is a rare complication, management guidelines are limited. However, there are several case reports discussing treatment options ranging from coil embolization, PTFE stent deployment and surgery [12–14].

## 2. Case Report

**Figure 1:**
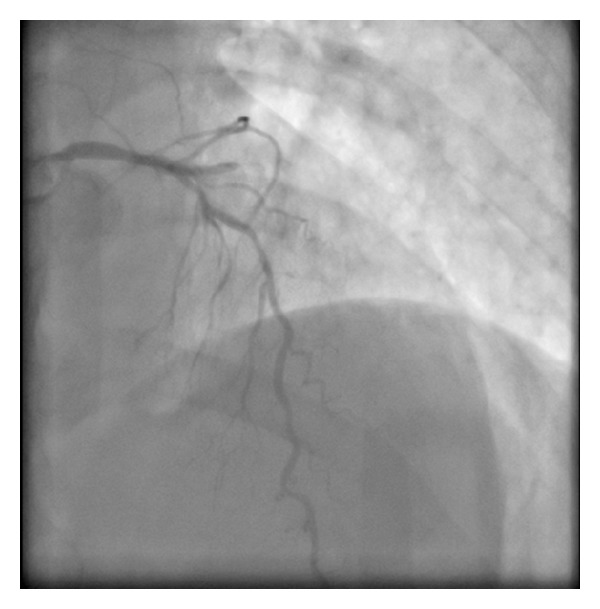
Diffusely diseased LAD with a 70% stenosis in the mid third with an occluded OM1.

**Figure 2:**
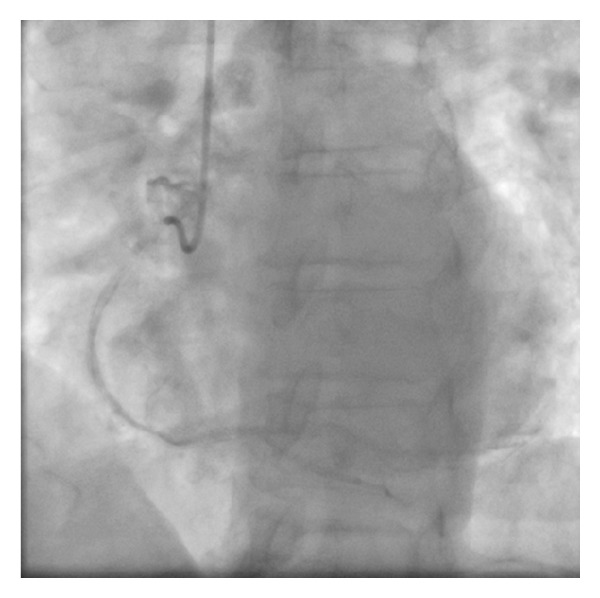
Nonselective injection of RCA.

**Figure 3:**
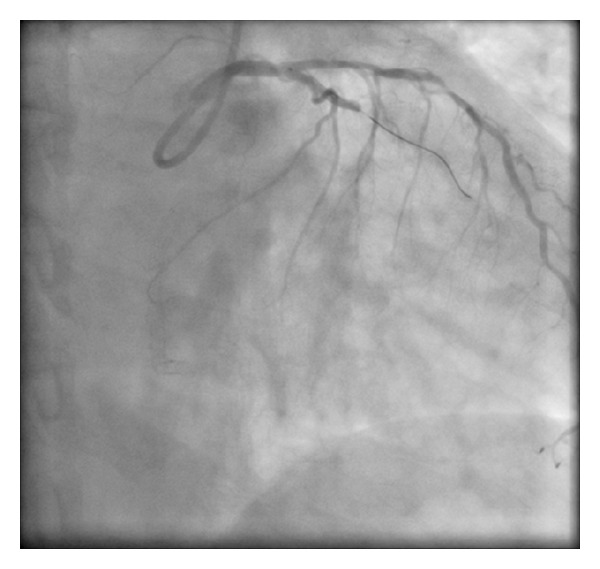
Crossing the LCX.

**Figure 4:**
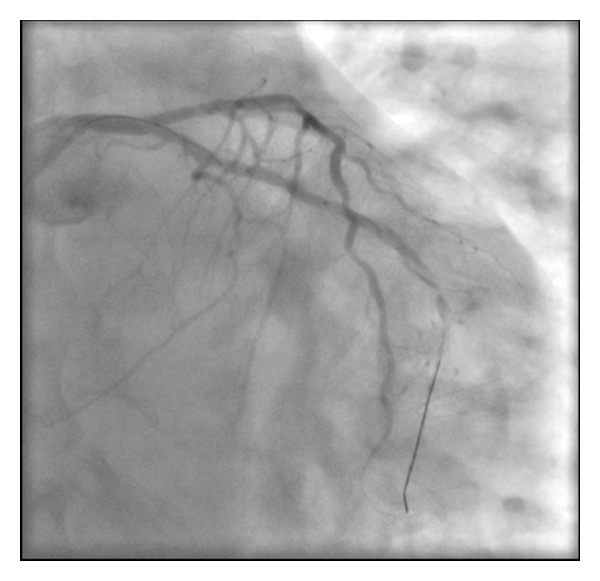
Type B dissection of proximal LCX.

**Figure 5:**
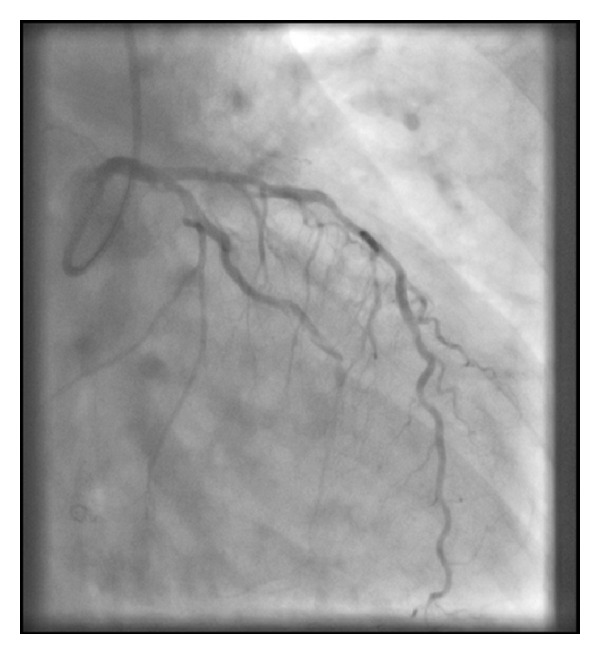
Type B dissection of mid OM1.

**Figure 6:**
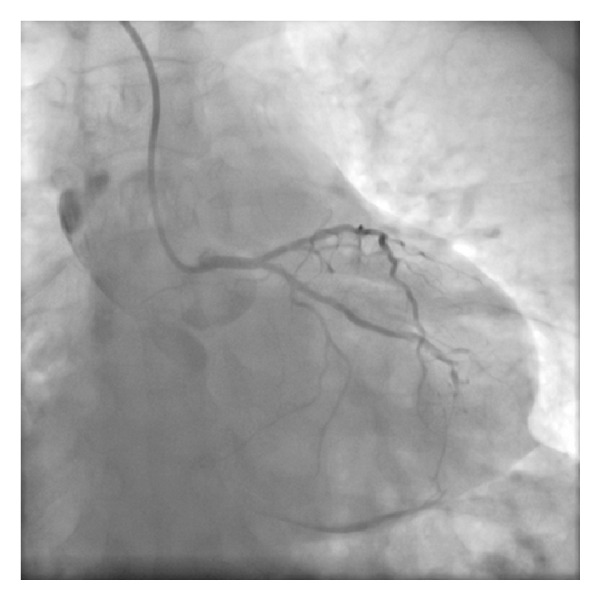
Coronary AV fistula (OM1 to coronary sinus).

A 61-year-old female presents with a non-ST elevation myocardial infarction (NSTEMI) in the setting of previous percutaneous coronary intervention (PCI) to the right coronary artery (RCA) with a bare metal stent (BMS) in 2004 on a background of treated hypertension and hypercholesterolemia and smoking. Her regular medications were aspirin 81 mg OD, metoprolol 25 mg BID, ramipril 5 mg OD and atorvastatin 40 mg OD. Since admission, she had ongoing chest pain. Her ECG had lateral ST-T changes and the peak cTnI was 3.0 ng/mL. She was brought emergently to the cardiac catheterization laboratory. The procedure was completed via right transradial catheterization. Coronary angiography showed diffusely diseased left anterior descending coronary artery (LAD) with a 70% stenosis in the mid third with an occluded first obtuse marginal coronary artery (OM1) with TIMI 0 flow (Figure 1). The RCA was anterior in origin and nonselective coronary injection showed a patent stent (Figure 2). The OM1 was considered to be the culprit vessel and attempted to cross with Pilot 50 wire (Guidant Corp., Indianapolis, IN, USA). The wire crossed the proximal subsection of the left circumflex (LCX) with support (Abbott TREK 2.0 mm × 15 mm coronary dilatation catheter) (Figure 3). There was a resultant type B coronary dissection of the mid subsection of the proximal LCX. A Wizdom Supersoft wire (Cordis Corp., Miami, FL, USA) was used to cross the dissection plane as a “parallel wire technique” to deal with the wire-induced dissection [15]. The proximal LCX was predilated with the 2.0 mm × 15 mm coronary dilation catheter to seal the proximal LCX dissection plane. Once flow was reestablished, the Pilot 50 wire and the 2.0 mm × 15 mm coronary dilation catheter were removed. The Wizdom Supersoft wire was used to advance down the OM1. A secondary type B coronary dissection was noted in the mid subsection of the mid third of the OM1 (Figure 4). The proximal LCX dissection plane appeared to have reduced flow and the dissection plane was again sealed with multiple balloon inflations. The patient remained pain-free with stable hemodynamics. The proximal LCX dissection plane appeared stable with TIMI 3 flow. Given the stable situation, the decision was made to stop. IV Eptifibatide infusion was started to stabilize the dissection plane with a plan to reattempt the CTO in 72 hours (Figure 5). The second procedure was also completed successfully via right transradial catheterization. Selective left coronary angiography showed a coronary artery (LCX) to coronary sinus fistula (Figure 6). The proximal LCX and the mid OM1 dissection planes appeared stable. As the patient remained pain-free and hemodynamically stable for 72 hours, the decision to manage medically with repeat noninvasive testing in 6–8 weeks was made. If there was evidence of reversible ischemia in the LCX territory (coronary steal syndrome), the decision would be to close the coronary AV fistula. Stress echocardiogram in 8 weeks showed no evidence of reversible ischemia in the LCX territory. The patient was advised to stay compliant with her current medications, continue dual antiplatelet therapy for a year, and make strong efforts to quit smoking.

## 3. Discussion

The challenge with managing coronary AV fistulas is due to the paucity of available management guidelines. This is due to the fact that it is a rare complication of PCI. However, the available management strategies are from several case reports and they range from observation, coil embolization, PTFE stent deployment and surgery [12–14]. As AV fistulas were large and caused coronary steal syndrome, the strategies were considered the best therapeutic option on the principle that they did not cause any harm to the patient and may be a potential benefit in the future.

It was lucky that the AV fistula was not large, and non-invasive testing 6 weeks later did not reveal any evidence of reversible ischemia or coronary steal syndrome. The strategy of observation worked for the patient and the probable reason for the absence of reversible ischemia or coronary steal syndrome is spontaneous resolution.
